# Navigating Foot Tuberculosis in a Leprosy Survivor: A Case Managed Solely With Medical Strategies

**DOI:** 10.7759/cureus.53991

**Published:** 2024-02-10

**Authors:** Hardik Patel, Aditya Pundkar, Sandeep Shrivastava, Saksham Goyal, Rohan Chandanwale

**Affiliations:** 1 Department of Orthopaedics, Jawaharlal Nehru Medical College, Datta Meghe Institute of Higher Education and Research, Wardha, IND

**Keywords:** tuberculosis osteomyelitis of the foot, leprosy, osteoarticular tuberculosis, extrapulmonary tuberculosis, foot tuberculosis

## Abstract

We present a case report of a 66-year-old male patient with a known history of leprosy who presented with pain and swelling in his right foot for the past 1.5 years. Fine needle aspiration cytology (FNAC) revealed non-inflammatory exudate, and *Mycobacterium tuberculosis* (MTB) was identified in the sample by the cartridge-based nucleic acid amplification test (CBNAAT). The patient was managed conservatively with anti-Koch's treatment (AKT), and a follow-up was conducted for 12 months to monitor the treatment response and overall progress. This highlights the importance of early diagnosis and appropriate medical management, along with a long-term follow-up, among patients with ankle tuberculosis, to reduce the need for surgical intervention.

## Introduction

Foot tuberculosis (TB) is a rare manifestation of extrapulmonary TB that can occur in individuals with or without a history of TB [1]. The prevalence of leprosy in India is 0.68/10,000 and the prevalence of TB is estimated to be 211/100,000 population for the year 2013 [2]. We report a case of foot TB in a patient with a concurrent history of leprosy, highlighting the challenges in its diagnosis and management. Leprosy, a chronic infectious disease caused by *Mycobacterium leprae*, has been a longstanding public health concern globally. Despite substantial progress in its management and control, individuals with a history of leprosy continue to face unique health challenges. In some cases, complications arise that demand careful consideration and specialized intervention. Foot TB, a rare manifestation of mycobacterial infection, poses a particularly intricate clinical scenario when it occurs in individuals with a background of leprosy. This case report delves into the exploration of such a challenging scenario, narrating the clinical journey of a leprosy survivor who developed foot TB and was managed exclusively through medical strategies. The intricacies of this case not only highlight the need for nuanced approaches in the management of coexisting conditions but also shed light on the potential effectiveness of medical interventions in navigating complex mycobacterial infections. Through a detailed examination of the clinical course and diagnostic challenges and the tailored medical management employed, this report aims to contribute valuable insights to the evolving landscape of infectious disease management, especially in individuals with a history of leprosy.

## Case presentation

A 66-year-old Hindu male patient working as a farmer presented with a complaint of pain and swelling in his right foot persisting for 1.5 years. The patient had a history of leprosy and was on regular medication (tab. rifampicin 600 mg once a week, tab. clofazimine 300 mg once a month and 50 mg daily, and tab. dapsone 100 mg daily) taken for six months for leprosy. For the past 1.5 years, he had been complaining of discomfort and swelling in his right foot, which led him to try several antimicrobial treatment regimens without success. Except for leprosy, his past medical history was not particularly noteworthy. There is no history of substance abuse, ethnicity, or stays in refugee camps, prisons, or night shelters. During a physical examination, the injured ankle showed signs of pain and induration. Additionally, it was clear that the range of motion of the ankle was restricted. Distal circulation was intact, sensation over the dorsum and plantar aspect of the foot was present, and no hypoesthesia was noted. Toe movement, as well as the power of the extensor hallucis longus (EHL)/extensor digitorum longus (EDL) and flexor hallucis longus (FHL)/flexor digitorum longus (FDL), was normal. Knee jerk, plantar reflex, and ankle reflex were within normal limits. No neural thickening was observed. His laboratory tests revealed abnormalities such as an increased erythrocyte sedimentation rate of 28 mm/h (reference: 2-10 mm/h) and a C-reactive protein level of 19 mg/L (reference: 6 mg/L). An elevated creatinine of 1.4 mg/dL (reference: -0.66-1.25 mg/dL), moderate anemia (hemoglobin=9 g/dL, reference value: 12-16 g/dL), human immunodeficiency virus, hepatitis B virus, hepatitis C virus, and brucellosis were all ruled out by the serologic analysis. The patient requested conservative management, so for sample collection, fine needle aspiration cytology (FNAC) was performed. FNAC of the affected ankle revealed non-inflammatory exudate, raising suspicions of an underlying infectious process. To confirm the etiology, a sample was collected and subjected to the cartridge-based nucleic acid amplification test (CBNAAT), which detected the low-level presence of rifampicin-sensitive *Mycobacterium tuberculosis* (MTB).

**Figure 1:**
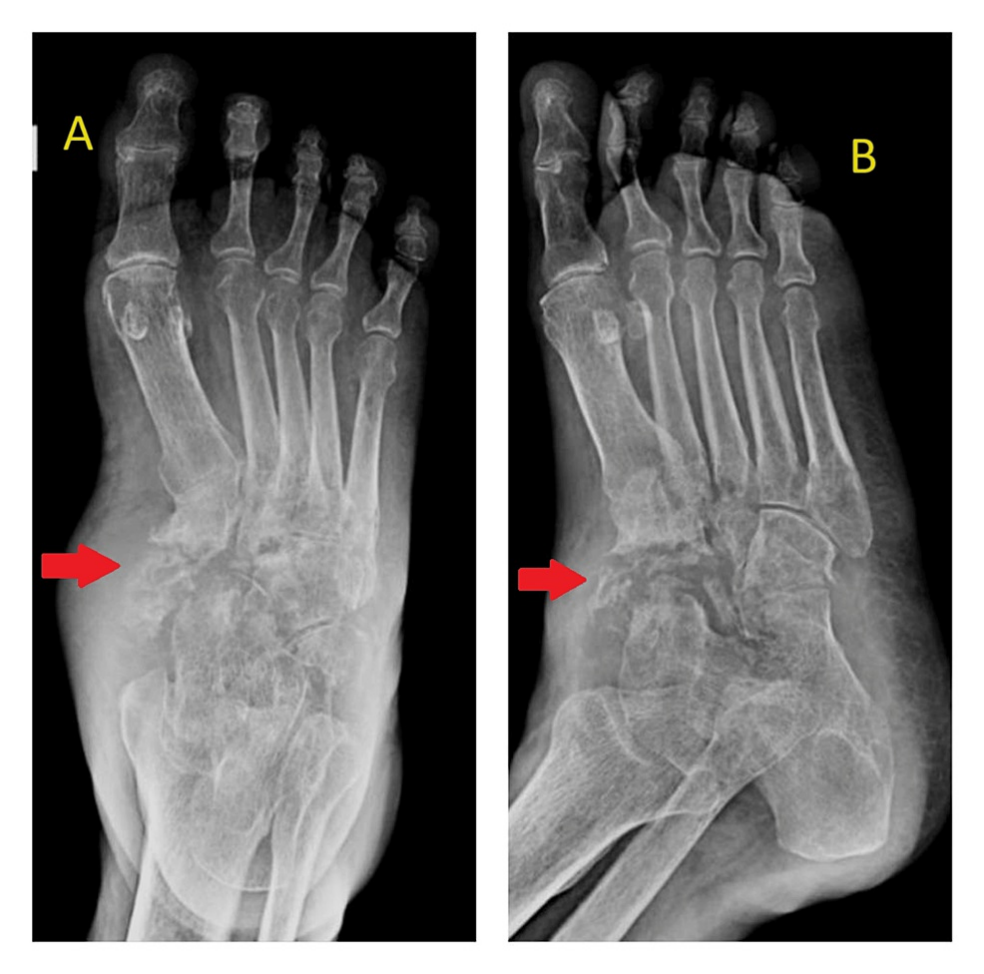
X-ray of right foot anteroposterior (A) and oblique (B) views showing generalized osteoporosis, periarticular soft tissue edema, subchondral erosions, or destructive lesions in the tarsal bones and base of the metatarsals suggestive of osteomyelitis changes; absence fracture seen.

**Figure 2:**
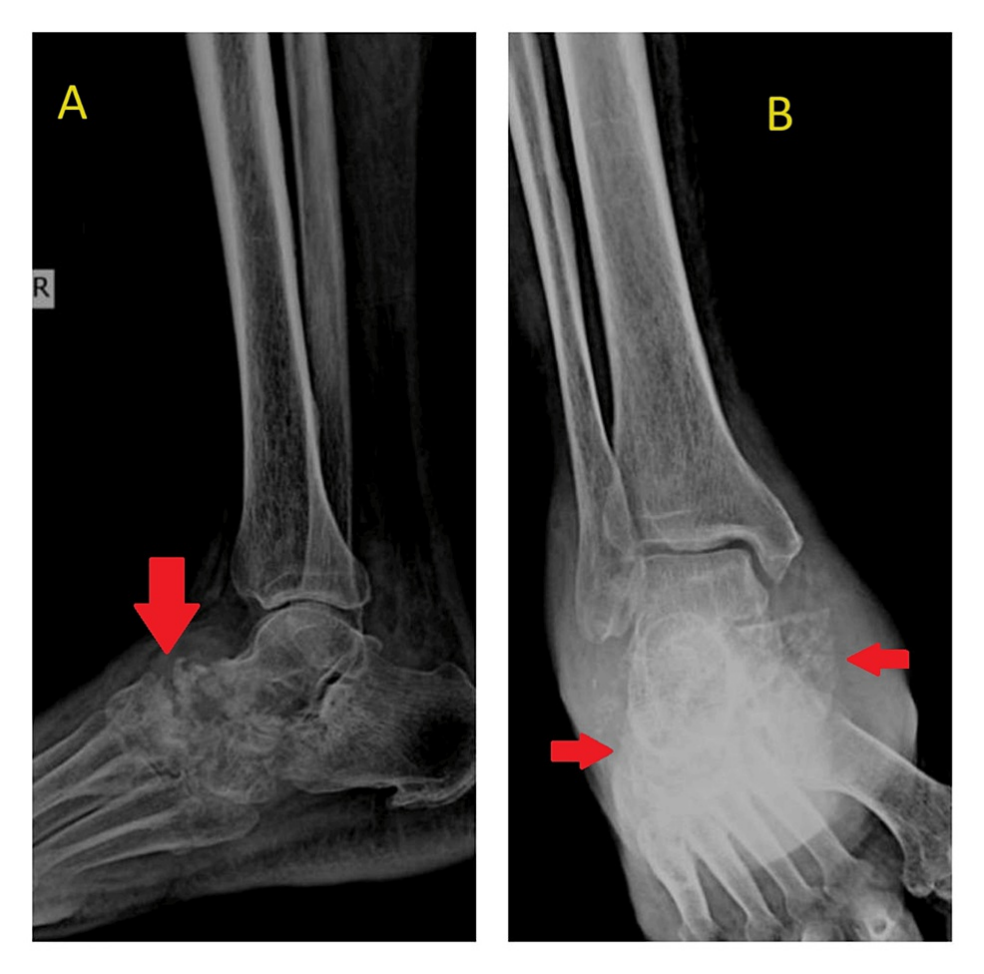
X-ray of right ankle lateral (A) and mortise (B) views showing osteomyelitis changes of the right intertarsal joints and tarsometatarsal joints.

The plain radiography (Figure 1 and Figure 2) revealed generalized osteoporosis, periarticular soft tissue edema, subchondral erosions, or destructive lesions in the tarsal bones and base of the metatarsals in the right ankle joint suggestive of osteomyelitis changes. Similar X-ray changes were also seen in Charcot arthropathy. Given the history of leprosy, there is a possibility of Charcot's disease. Charcot arthropathy is typically associated with neuropathy, especially diabetes mellitus, and is characterized by a history of repetitive microtrauma or chronic pressure on insensate feet. It tends to be relatively painless or may cause mild discomfort due to neuropathy. In contrast, tuberculous osteomyelitis is marked by soft tissue involvement, abscess formation, and swelling, accompanied by significant localized pain. Differentiating between these conditions relies primarily on history and physical examination, as radiologically, they may not be easily distinguishable.

Following a consultation with an orthopedic surgeon, an MRI of the afflicted foot and articular FNAC guided by ultrasound were advised because there may be chances of bacterial osteomyelitis, tuberculous osteomyelitis, Poncet's disease, or Madura foot. Although the patient did not want an MRI due to financial issues, he did agree to have tissue samples taken. A non-inflammatory exudate was discovered by FNAC on the affected foot, indicating the possibility of an underlying infectious disease. A sample was taken and submitted to CBNAAT, which identified the presence of MTB, to validate the etiology. A dose-adjusted quadruple anti-TB regimen, consisting of isoniazid 5 mg/kg daily, rifampicin 10 mg/kg daily, ethambutol 15 mg/kg daily, and pyrazinamide 20 mg/kg daily, was also started for the patient. At the nine-month follow-up, his discomfort and edema had greatly diminished. Considering the patient's history of leprosy and the positive CBNAAT result, a conservative management approach was adopted. The patient was initiated on anti-Koch's treatment (AKT), a standard TB treatment regimen comprising isoniazid, rifampicin, ethambutol, and pyrazinamide, for two months followed by isoniazid, rifampicin, and ethambutol for 10-18 months [3]. Close monitoring and follow-up were scheduled to assess the treatment response and evaluate any adverse effects.

**Figure 3:**
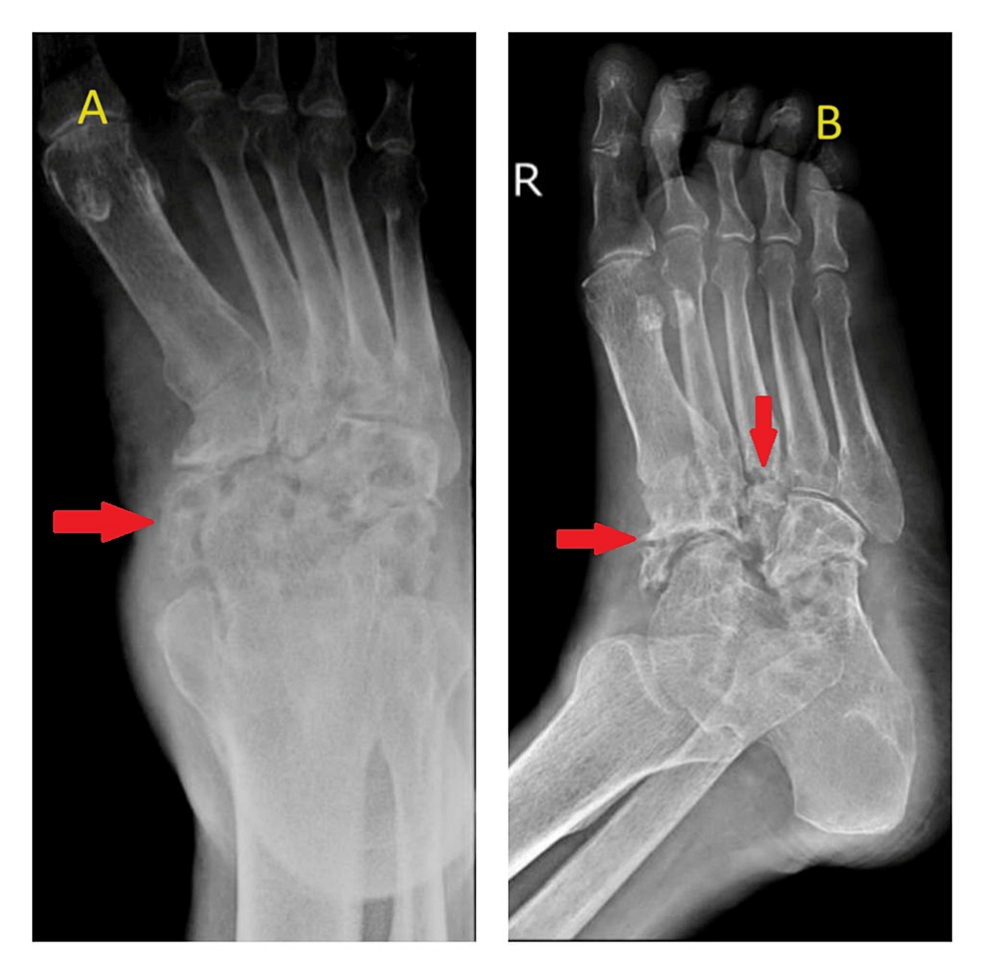
X-ray of right foot anteroposterior (A) and oblique (B) views showing lytic lesions noted in the tarsal bones. No bony erosion or destruction was noted. The joint space appeared normal. No other osteomyelitis changes were seen.

**Figure 4:**
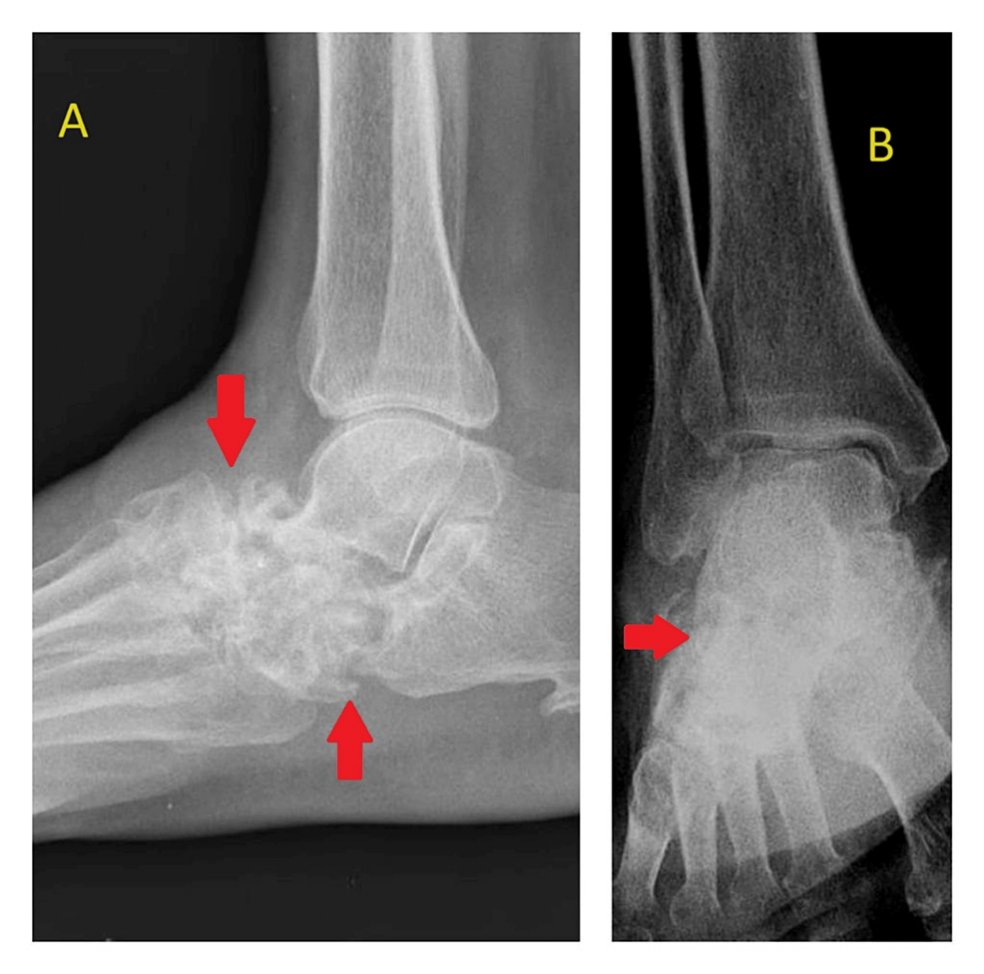
X-ray of right ankle lateral (A) and mortise (B) views showing only lytic lesions noted in the tarsal bones. No other osteomyelitis changes were seen.

During the 12-month follow-up period, the patient's clinical condition gradually improved. The pain and swelling subsided, and the patient was subjectively relieved with uneventful recovery. Periodic laboratory investigations, radiographic imaging, and physical examinations were conducted to monitor the response to treatment and rule out any complications. After the 12-month follow-up period, laboratory investigations such as hemoglobin of 11 g/dL, erythrocyte sedimentation rate of 8 mm/h, and C-reactive protein of 5.8 mg/L were found to be normal. An ankle-foot orthosis was provided for deformity correction, to prevent ankle stiffness, and to facilitate early mobilization. After the 12-month follow-up period, the plain radiography (Figure 3 and Figure 4) revealed only lytic lesions noted in the tarsal bones. No bony erosion or destruction was noted. The joint space appeared normal. No other changes suggestive of osteomyelitis were observed.

## Discussion

Foot TB is an uncommon presentation, and its diagnosis can be challenging due to its similarity with other conditions such as chronic osteomyelitis or Charcot arthropathy. FNAC may help establish the inflammatory nature of the lesion, while CBNAAT provides definitive evidence of MTB infection [4]. Similar diagnostic dilemmas are encountered in TB arthritis, where clinical suspicion often overlaps with other forms of arthritis or infectious processes. While FNAC and CBNAAT aid in confirming TB, a synovial biopsy and an acid-fast bacteria test on the synovial fluid are essential for definitive diagnosis [5]. The importance of differentiating TB arthritis from reactive or septic arthritis is emphasized, as misdiagnosis can lead to treatment delays and potential complications. Management typically involves administering appropriate anti-TB medications, as in this case, AKT. The etiology of TB arthritis is either an infection that spreads from a close bone or a hematogenous spread from the lungs [6]. Extrapulmonary TB generally appears in immunosuppressed people with human immunodeficiency virus, diabetes mellitus, leprosy, cancer, or alcoholism or those who are being treated with corticosteroids or immunomodulators. Additionally, acute injury due to trauma, surgery, or the use of intravenous drugs may cause TB to reactivate in the nearby joints [7]. However, it is possible that none of the aforementioned ailments exist in endemic regions. MTB causes arthritic involvement by a slow, sneaky process that initially manifests as simple synovitis and is reflected in increased joint space in X-ray; granulation tissue, effusion, and cartilage deterioration thereafter develop. The underlying bone may become impacted in the subsequent stage, or para-articular cold abscesses may develop, leading to the creation of fistulae and a sinus drainage tract [8]. Monoarticular patterns are typical in the presentation of TB arthritis. Peripheral TB arthritis most usually manifests in the hip and knee [9]. However, some patients exhibit ankle or foot joint involvement [10].

TB should be considered a potential differential diagnosis in all subacute to chronic arthritis. In cases of arthritis involvement and a nasal tube draining to the skin above, this is quite obvious [11,12]. As was the case with our patient, TB arthritis is frequently misdiagnosed as reactive or septic arthritis and treated as such, which delays the detection of the condition [12]. To confirm the diagnosis, a synovial biopsy and an acid-fast bacteria test on the synovial fluid should also be performed [13]. A granulomatous synovitis may indicate TB [13]. Our patient nevertheless was started on anti-TB medication because the diagnosis of TB arthritis was primarily based on clinical suspicion and the results of the FNAC and CBNAAT reports. The Xpert MTB/RIF assay and culture can also be used to determine the isolate's antibiotic susceptibility [14]. The X-ray results of our patient's foot were suggestive of generalized osteoporosis, periarticular soft tissue edema, subchondral erosions, or destructive lesions in the tarsal bones and base of the metatarsals in the right ankle joint suggestive of tuberculous osteomyelitis changes, and the histology results of his joint sample supported our diagnosis. Suspects of TB arthritis can also benefit from imaging techniques. Clinicians cannot detect early articular involvement with plain radiography. Only in the advanced phases of TB arthritis are juxta-articular osteoporosis, peripheral osseous erosions, and increasing joint space narrowing visible [15]. Nevertheless, these symptoms were visible on our patient's plain radiography due to the delayed diagnosis. Sadly, our patient declined to get an MRI. However, the utilization of advanced imaging techniques like MRI is crucial for detecting early articular involvement and concomitant abnormalities in TB arthritis. MRI provides superior visualization of soft tissues, joint effusion, bony erosions, loose bodies, and calcifications facilitating accurate diagnosis and treatment planning [11]. Finally, pharmaceutical therapy and, in certain cases, surgical treatments are part of the treatment plan for TB arthritis. The standard four-drug regimen is used as part of medical therapy for at least 12-18 months [3]. However, a prior study showed that medical management of TB arthritis typically involves a prolonged course of anti-TB medication, often supplemented by surgical interventions such as bone debridement to address extensive bony involvement or complications like abscess formation. Using anti-TB medications along with bone debridement could greatly enhance the patient's prognosis [16]. Long-term follow-up is essential in TB arthritis to assess treatment efficacy, monitor for disease recurrence, and manage potential sequelae such as joint deformities or functional impairments. Multidisciplinary care teams play a crucial role in providing comprehensive patient management throughout the treatment course [17].

## Conclusions

This case underscores the complex challenges in managing foot TB in leprosy survivors, highlighting the efficacy of tailored medical approaches. The successful outcome showcases the evolving landscape of infectious disease management, emphasizing the importance of nuanced strategies. Diagnostic complexities and therapeutic success underscore the need for refined approaches in similar cases, contributing to our understanding of mycobacterial infections in leprosy survivors. This case emphasizes the importance of considering foot TB in patients with chronic foot pain and swelling, especially those with a history of leprosy or TB. Early diagnosis, utilizing techniques like FNAC and CBNAAT, enables prompt treatment initiation and favorable outcomes with a conservative four-drug regimen for at least nine months. Regular follow-up is crucial for monitoring treatment response, adjusting the regimen if needed, and preventing complications.
